# Understanding Mental Health Through the Theory of Positive Disintegration: A Visual Aid

**DOI:** 10.3389/fpsyg.2019.01291

**Published:** 2019-06-04

**Authors:** Marie-Lise Schläppy

**Affiliations:** Faculty of Engineering and Mathematical Sciences, Oceans Graduate School, The University of Western Australia, Perth, WA, Australia

**Keywords:** theory of positive disintegration, depression, anxiety, overexcitabilities, K. Dabrowski, mental health, neurosis

The theory of positive disintegration (TPD) is a complex theory of personality development elaborated by K. Dabrowski (1902–1980). The characteristics of this theory is that some signs of mental illness (e.g., neurosis, anxiety) along what is often considered a person's flaws (e.g., nervousness, maladjustment) are seen as positive signs that a person is developing their personality toward their “personality ideal” (i.e., the best, most altruistic, and worthy version of themselves) (Dabrowski, 1964; Dabrowski and Joshi, 1972). The implications of the TPD are that symptoms of poor mental health may not always be negative, but part of a necessary process which lets individuals who successfully navigate those difficult inner-states grow to be the best version of themselves.

In the TPD, the path to personality development is expressed as a series of levels represented by the Roman numerals I–V (Figure 1). To explain how a person might be undergoing personality development, dynamisms (e.g., disquietude with oneself, subject-object in oneself) are used to explain the feelings of a person when they transit from one level to another (Dabrowski, 1964) (Figure 1). The theory stipulates that neither ontogeny nor intelligence influences the level at which a person will find themselves. The potential of any individual to develop their personality relates to three factors: (1) genetic attributes, also called overexcitabilities (psychomotor, sensual, imaginational, intellectual, and emotional) (2) the environment (3) the inner motivation to develop one's personality (called the third factor) (Dabrowski et al., 1972).

**Figure 1:**
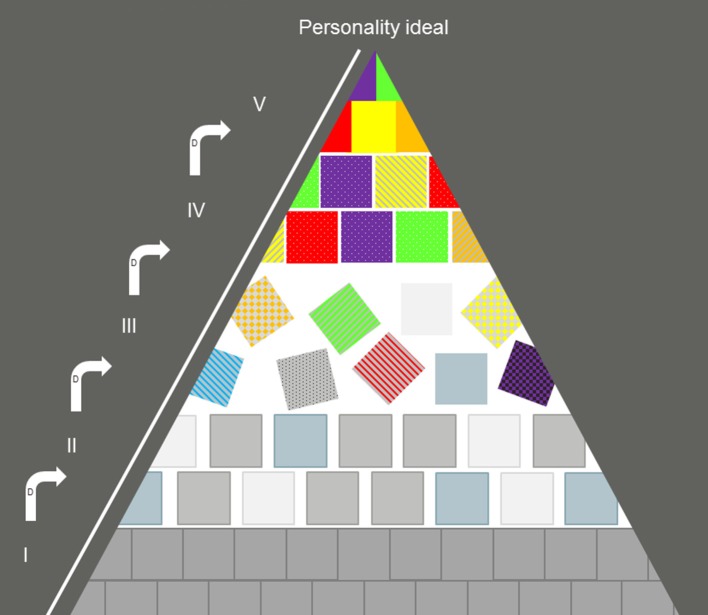
Graphical representation of the five levels of the theory of positive disintegration. Level I: primary integration, Level II: uni-level disintegration, Level III: spontaneous multi-level disintegration, Level IV: directed multi-level disintegration, and Level V: secondary integration. D, dynamisms which are the mechanisms by which a person switches from one level to another. The proportions of the pyramid are not meant to be quantitatively accurate. If we consider that the base of the pyramid should be representing 70% of the total base of the sections, the pyramid should really be squatter. However, when drawn it that fashion, it becomes difficult to illustrate level V adequately so this schematic representation of levels is therefore a compromise.

The theory has a following in the realm of gifted education where some components of the theory (overexcitabilities) are sometimes used to identify gifted pupils. To date, empirical evidence showing how overexcitabilities correspond (or not) to known psychological constructs is scant (but see Rinn and Reynolds, 2012; Vuyk et al., 2016). Attention has been drawn to the risk of using the concept of overexcitabilites outside the context of the whole TPD theoretical framework (Mendaglio and Tillier, 2006), as overexcitabilities are only one (the first factor) of three factors influencing personality development. The TPD is generally poorly known among psychologists, psychiatrists, and (mental) health practitioners and as it is rarely part of formal vocational or university training. Yet the TPD offers a novel view of mental health (Dabrowski and Joshi, 1972) that may have its place in helping patients work through their mental health-related challenges and will help therapists understand their patients. There is anecdotal evidence to suggest that mental health practitioners find the TPD invaluable when counseling children and adults who show signs of what is commonly viewed as poor mental health (e.g., anxiety, neurosis, etc.) but also when counseling patients who show other characteristics that may decrease their well-being (e.g., perfectionism, social maladjustment, etc.).

Adopting the view that the theory has merit, an attempt is made here at providing a visual representation of some of its key aspects with the aim of providing a memory aid to help its understanding and dissemination. This diagram could help theoreticians, psychometricians, and practitioners to understand some fundamental concepts of the theory beyond its better-known aspects (overexcitabilites). The concepts presented here will hopefully be intriguing enough to prompt the reader to seek original writings by K. Dabrowski for a full understanding of this theory and its value (see Dabrowski, 1964, 1967, 1972; Dabrowski et al., 1970, 1972).

A word of caution is probably appropriate for those who want to start studying the main concepts of the theory: K. Dabrowski often gives a new, unique meaning to words commonly used in the field of psychology. This may cause irritation to specialists and may act as a barrier to further study of the theory. Novices to the field or laypersons may find less reluctance to accept the alternative meaning given to some words in the TPD. To those who do not pass this initial barrier, the theory might just appear to be the product of a confused scholar, essentially not worth studying. However, persistent study of the TPD does reveal its depth and intricacies and offers a quite different and interesting view on mental health. This paper aims to introduce theorists and practitioners to the TPD, and for this reason, a lexicon of key terms is provided (Box 1).

Box 1 **Meaning of key terms in the theory of positive disintegration.**

**Dynamisms:** Mechanisms by which individuals are propelled, or propel themselves, from one level of personality development to another. Dynamisms are often formulated as feelings (e.g., astonishment, dissatisfaction with self, or empathy) but now always (e.g., creativity, self-awareness).

**First factor:** A combination of 5 characteristics that some individuals possess, also named overexcitabilities (OEs) which results in an increased discrepancy between the qualitative experience of life of an individual compared to the social settings in which that individual finds themselves. There are 5 overexcitabilities: psychomotor, sensual, emotional, intellectual, and imaginational (neologism used by K. Dabrowski to signify “which stems from the imagination,” and stands in contrast to “imaginary” (i.e., “not real”)). The OEs are often used in the field of gifted education. The stronger they are the more likely an individual will experience inner conflicts that may result in positive disintegration.

**Multi-level:** A person who has reached a multi-level view of the world is one that see “what is” and “what should be” in terms of an individual's values and keenly feels the difference between the two (Level III, IV, and V in Figure 1). Individuals with strong overexcitabilities have a higher probability of gaining a multi-level view and therefore to find themselves at level III, IV, or V.

**Overexcitabilities:** See “first factor.”

**Personality:** A state of development that is attained when an individual, works toward and reaches their personality ideal.

**Personality ideal:** The best, most noble version of a person specified by themselves with characteristics that all would recognize as noble and benefiting the greater good.

**Positive disintegration:** The state represented by level III (Figure 1), in which the personality of an individual is transitioning from being based on values given by biological factors (the need to eat, seek shelter, and reproduce) and by social factors (gaining social standing, belonging to a social group whose values are adopted without critical scrutiny) toward self-determined values geared toward the greater good.

**Second factor** (environmental factor): the external circumstances that an individual find themselves in, which foster or impede their trajectory toward reaching their personality ideal.

**Third factor:** The inner urge to better oneself, to work toward ones personality ideal that stems from intrinsic motivation.

**Unilevel:** A person who is unilevel (level I and II in Figure 1) does not perceive any difference between “what is” and “what should be”, because to them, they are the same.

The TPD is a theory of personality development. Many call it a theory of “emotional development” and although emotions are at the heart of the theory, the TPD is essentially centered on the concept of personality and of the “personality ideal” (Dabrowski et al., 1972). For K. Dabrowski, the word “personality” does not refer to individual human characteristics present since birth or gained through exposure to one's environment. Dabrowski's premise is that not only are we born without a personality but many individuals never develop one. Gaining a personality, according to the TPD, involves striving toward one's personality ideal (Dabrowski, 1964). Those who fail to undertake that journey, simply remain without a personality all their life. The personality ideal (Figure 1) is unique to each individual but is essentially the best possible version of oneself that one may attain in a lifetime. This definition of personality stands in stark contrast with a more standard definition of personality, where it is often thought to be the “the quality or state of being a person”^1^, which shapes itself since (or even prior to) birth, is possessed by all humans, and is gained principally without conscious effort. For these reasons, care should be taken when comparing the TPD to other theories of personality development because the word personality refers to two very different concepts that cannot be readily compared.

The TPD predicts that those who undertake the journey toward gaining a personality (by progression toward their personality ideal) may find themselves at four different levels (II–V) (Figure 1). Those who do not undertake this journey typically stay at level I (Figure 1). Unlike consecutive stages (such as for example, Maslow's pyramid of needs), the levels in the TPD are not sequential. In other words, one does not start at level I and journey to level V. Rather, a person may be at a certain level at a specific point in time and the TPD model gives a prediction of the mechanisms (dynamisms) and likelihood of that person going to another level. In Figure 1, the levels are represented in a pyramid because Dabrowski hypothesized that only very few individuals are at level V, whereas a majority probably is at level I, hence the broad base of the geometrical shape. Anecdotal evidence suggests that K. Dabrowski thought that level I would be a fair representation of 70% of the human population (see Piechowski, 2016, p. 12), although this estimate has never been published in his own work.

At level I, also called primary integration, an individual's values are essentially based on their biology and membership to social groups. The values of an individual of level I are determined by the need to fulfill their biological needs (eat, sleep, reproduce, and avoid harm) and social needs (behave in ways that are acceptable to the social groups the individual belongs to and gain status within the groups). The undeniable advantage of level I is the predicted lack of inner-conflict (depicted in Figure 1 by the lack of color white). The bricks in Figure 1 represent the values of a level I individual, which are uniform and firmly set together, forming a very stable structure, and correspond to biological and social values. Some scholars interpret level I as being very negative (e.g., the level of psychopaths). This view is unhelpful because a theory that proposes that 70% of the population are psychopaths cannot be taken seriously. As pointed out by Mendaglio and Tillier (2015), Dabrowski stated that psychopaths are only a subtype of people found at level I (Dabrowski, 1964). From now on, I propose to view level I as persons who, although they may not develop a personality, possess stability, consistency and predictability of behavior through the lack of inner conflict (represented by the color white on Figure 1). This view does not seem to contradict with Dabrowski's description of level I and offers a way for most of us to identify better with that level. Combined with desirable character traits like kindness and good will, persons at level I can provide the much-needed support and stability that the people at non-integrated levels lack (levels II–IV). Persons at level I may fundamentally be unable to empathize with those who experience disintegration, but may be supportive and tolerant nonetheless, while at the same time be driven only by values relating to their biology and their environment. I suggest that they can provide a stable, well-meaning and kind presence in the lives of those individuals at less integrated levels and propose, therefore, that the value of level I rests in its stability and predictability.

The traditional view of Level II [but see Tillier (2009) for an explanation of differing views] is that it is a transitory level, in which an individual starts experiencing inner-conflict (white slivers between the colored bricks in Figure 1). At level II an individual cannot consciously move toward level III because their view of the world is unilevel (i.e., they do no apprehend that their values could change significantly and that “what is” and “what should be” are not necessarily the same thing). Just like at level I, individuals at level II have values that align with biology and social/environmental context, but they might start changing slightly (see, different gray shades of the bricks on Figure 1) due to the emergence of inner conflict. An individual at level II will suffer from the unconscious inner conflict to such an extent that they will relatively quickly revert to level I or tip over uncontrollably into level III (Dabrowski et al., 1972). Dabrowski (1964, 1967) hypothesized that if a person was trapped at level II, they may experience schizophrenic symptoms or may be in danger of committing suicide because of the weight of unconscious inner conflict (slivers of white on Figure 1), ambivalence and ambitendance on the psyche.

At level III, the level of spontaneous multilevel disintegration, an individual has the ability to differentiate “what is” and “what should be,” thereby gaining multi-level perception (Dabrowski, 1964). At that level, an individual is able to self-reflect and determine when they are acting according to level I or II values or when they are acting according to self-made values (gray/colored bricks on Figure 1). Conscious inner conflict ensues (large areas of white at level III on Figure 1). Acquiring multi-levelness may not be a controlled process but might be rather spontaneous (bricks (= values) going in all directions (Figure 1)). Some values remain the same as before (bricks with gray tones) and some change drastically toward new, self-chosen values that may be quite to different the values of level I and II (textured, brightly colored bricks, Figure 1). An individual at this level might be experiencing symptoms such as anxiety, and neurosis but also has the awareness necessary to actively develop their personality in a self-directed, autonomous way, toward their personality ideal (Dabrowski, 1964, 1967; Dabrowski et al., 1972).

It follows that practitioners who have patients at this level will have a large role to play toward fostering their personality development. They can either accompany the person toward further development to reach level IV where inner conflict is reduced and values have taken a very personal color, or, their action can thwart a person's developing personality and push them back toward level II and ultimately level I. The theory stipulates that the disintegration of those values is a positive development in the life of an individual, therefore the name of “positive disintegration” (Dabrowski et al., 1972). It follows that although patients in level III can show a high degree of inconsistent behavior, crises and mental health symptoms, they are, more than any other levels, in a position to benefit from an informed, supportive counseling intervention. That is because, if a person is supported rather than thwarted in their development, they might reach level IV, where the person's inner values have taken their own decisive colors despite some remaining inner-conflict (Figure 1). Practically, this would be exemplified by people who can act according to their own values in most situations, except when external circumstances are not conducive or when self-doubt assails them. Knowledge that level III is only transitory may help patients with depression and anxiety manage their negative affect, which, according to the theory of personality of systems interaction is hampering one's ability to self-regulate and motivate (Kuhl, 2000). Future research may show that mental health practitioners can have a large role to play in accompanying their clients though the transition from level III to level IV by sharing the posits of the TPD with their clients.

At level V a person can act according to self-developed values all the time, and under all circumstances and those values are turned toward the greater good rather than self-serving goals, which are values typically seen at level I. Like persons at level I though, an individual's actions at level V are predictable in their constancy. At level V individual has reached their personality ideal. Figure 1 shows only a very small and narrow part of the pyramid for level V, as it is hypothesized that the level is attained by only a minority of the population, although there are few easily accessible and peer-reviewed empirical evidence to date to support this claim; but see Mendaglio (2008) for examples.

The TPD's strengths are that, instead of considering persons with mental issues as sick (neurosis, anxiety, etc. see original Dabrowski works for an extensive list), the theory proposes that it is the very persons who show those symptoms who have the highest potential for growth and personality development. The strengths of the TPD clearly lie in the different view it gives of certain mental health symptoms. The corollary is that those individuals, who show symptoms of ill mental-health, may be the very individuals who may ultimately be able to make a disproportionate contribution to the greater good if they are supported in their inner development. Equally, they will be the same individuals who may become a burden to society if they are made to fit into a world where their newly found inner values have no place. In terms of public health, the TPD would postulate that investing in those patients is a sound investment for society. The limitations of this theory is the limited empirical evidence to support the theoretical framework outside the gifted education field, the field in which it has been traditionally used, and the corresponding emphasis on the first factor, the overexcitabilities. Other limitations are that some mental health problems such as schizophrenia are unlikely to represent signs of positive disintegration. Moreover, the validity of this theory should be further applied in cross-cultural studies to test its universal validity.

In summary, this visual tool may help disseminate some of the basic concepts of the TPD, a theory of personality development that will hopefully now receive more attention by theoreticians and practitioners alike. By considering persons in disintegration through the lens provided in the TPD, mental health practitioners could help those persons toward a positive, higher version of themselves. The TPD offers such an alternative view of mental health that all who are in contact with persons in disintegration should be acquainted with the fundamentals of the TPD.

## Author Contributions

M-LS invented the visual aid and wrote the paper.

### Conflict of Interest Statement

The author declares that the research was conducted in the absence of any commercial or financial relationships that could be construed as a potential conflict of interest.

## Footnotes

^1^https://www.merriam-webster.com/dictionary/personality
